# Useful functional recovery and quality of life after surgical treatment of peroneal nerve injuries

**DOI:** 10.3389/fsurg.2022.1005483

**Published:** 2022-11-14

**Authors:** Lukas Rasulić, Živan Nikolić, Milan Lepić, Andrija Savić, Filip Vitošević, Nenad Novaković, Stefan Radojević, Aleksa Mićić, Sanja Lepić, Stefan Mandić-Rajčević

**Affiliations:** ^1^ Faculty of Medicine, University of Belgrade, Belgrade, Serbia; ^2^ Clinic for Neurosurgery, Clinical Center of Serbia, Belgrade, Serbia; ^3^ Clinic for Plastic Surgery and Burns, Military Medical Academy, Belgrade, Serbia; ^4^ Clinic for Neurosurgery, Military Medical Academy, Belgrade, Serbia; ^5^ Center for Radiology and MRI, Clinic for Neurosurgery, Clinical Center of Serbia, Belgrade, Serbia; ^6^ Medical Faculty of the Military Medical Academy, University of Defence, Belgrade, Serbia; ^7^ Institute of Hygiene, Military Medical Academy, Belgrade, Serbia; ^8^ School of Public Health and Health Management and Institute of Social Medicine, Faculty of Medicine, University of Belgrade, Belgrade, Serbia

**Keywords:** peroneal nerve, trauma, outcome, quality of life, surgery

## Abstract

Closed injuries to the peroneal nerve recover spontaneously in about a third of patients, but surgery may be needed in the remaining 2/3. The recovery after surgery is not always satisfactory and the patients may need an orthosis or a walking aid to cope with regular daily activities. This study aimed to evaluate the useful functional recovery and quality of life (QoL) in surgically treated patients with peroneal nerve (PN) injuries. The study involved 51 patients who have undergone surgical treatment due to PN injury in our department, within a 15-year period (2006–2020). Thirty patients (59%) were treated with neurolysis, 12 (23%) with nerve repair techniques, and 9 (18%) with tendon transfer (TT). Neurolysis is employed in the least extensive nerve injuries when nerve continuity is preserved and yields a motor recovery ratio of almost 80%. Nerve repairs were followed by 58.33% of patients achieving M3+ recovery, while 41.66% recovered to the useful functional state (M4 or M5) With the use of TTs, all patients recovered to the M3+, while 66.7% recovered to M4. All our results correspond to the results of previous studies. No statistically significant differences were found regarding the QoL of the groups. There is an apparent advantage of neurolysis, over nerve repair, over TT procedure, both in terms of useful functional recovery, and foot-drop-related QoL. However, when involving all aspects of QoL, these advantages diminish. The individual approach leads to optimal results in all groups of patients.

## Introduction

Peripheral nerve injuries account for 2%–3% of all patients admitted to primary-level trauma centers (1). According to the previously published data, they are present in less than 2% of patients with limb injuries, while less than 1% of cases involve a peripheral nerve injury in the lower extremity (2). Although rare, the injuries may have a devastating impact on all aspects of living and significantly decrease quality of life (QoL) (3).

Peroneal nerve (PN) palsy presented as “foot drop”, is the most common mononeuropathy, but the majority of cases are related to radiculopathy and herniated discs, with favorable natural history and treatment outcomes (4), and rarely entrapment neuropathy or injury (5). Closed injuries to the PN may recover spontaneously, without specific treatment, in about a third of patients, but in the remaining 2/3, a permanent foot drop remains (6, 7). These patients are usually candidates for surgery, followed by a prolonged period of physical treatment and methods for the augmentation of nerve recovery in a multidisciplinary setting (8). The recovery is not always sufficient, and even M3 according to the Medical Research Council (MRC) scale for muscle strength (9), may end up as insufficient for some patients. Sometimes, a traditional or high-tech orthosis or walking aid may be needed to cope with regular daily activities (10).

Surgical options to treat these patients include procedures aimed at the exploration and various forms of neurolysis (external, internal, intrafascicular) (11), and nerve repair options (direct repair, grafts, or artificial conduits) (12, 13). Nerve transfers do not have the same beneficiary effect in the lower as they do in the upper extremity (14, 15), while tendon transfers (TTs) may be applied with success when recovery capacity is compromised (16, 17). Previous studies reported different results of motor recovery with the use of these techniques (18), and their combined use efficacy (19).

Motor recovery is the most commonly measured outcome, and there are some tools to asses useful functional recovery and QoL, however, with some applicable limitations. Patients treated for lower extremity nerve injuries were rarely evaluated in light of QoL before, while the foot drop is usually evaluated in terms of QoL with a focus on the need for braces, most commonly with the Stanmore system (20, 21).

This study aimed to evaluate useful functional recovery and QoL in surgically treated patients with PN injuries, as well as the evaluation of chosen general QoL inventories in terms of lower limb and PN affection.

## Methods

The study involved a retrospective series of patients who have undergone surgical treatment due to the PN injury at our department in a 15-year period from January 2006 to December 2020.

All procedures performed were in accordance with the institutional ethical standards and with the 1964 Helsinki declaration and its later amendments. Informed consent was obtained from all individual participants included in the study.

### Inclusion criteria

• Patients with surgically treated PN injury
• Traumatic and iatrogenic nature of injury
• Common stem or branches involvement

### Exclusion criteria

• Bilateral PN injury
• Associated tibial nerve injury

Out of 57 surgically treated patients fulfilling the inclusion criteria, six patients were excluded: one due to the bilateral injury, and five due to the associated tibial nerve injury.

### Management

Within the preoperative evaluation, patients and injury characteristics were recorded, including the details on previous surgical interventions and associated injuries. Muscle strength was evaluated using the MRC, sensibility of the affected region using the Mackinnon–Dellon scale (MDS) (22), and the visual-analog scale (VAS) for the assessment of pain.

The individually tailored approach and decision-making on the modality of surgical treatment depended on two important features: (1) nerve continuity and (2) reinnervation capacity; based on the clinical and neurological examination, electrophysiology, imaging, and the time passed from injury to our initial examination.

Neurolysis was performed in patients with preserved continuity and reinnervation capacity (preserved efferent muscles); nerve repair with graft when the nerve was interrupted in continuity, but with preserved reinnervation capacity; and TT when there was no reinnervation capacity (usually due to the long-standing nerve lesion), regardless of the continuity.

The right timing is of utmost importance in peripheral nerve surgery. Immediate repair may be performed in open injuries with clear cuts. Open injuries involving the proximal and/or distal ends (e.g., laceration), are repaired in a delayed fashion, usually 3–4 weeks after the injury, while the initial surgery usually includes identification of the proximal and distal roots and their marking. In closed injuries, electrophysiological studies to confirm the lesion are carried out 3–4 weeks after the injury, but surgical treatment is indicated only in cases with no signs of recovery in electrophysiological studies performed 12 weeks after the injury (13). TTs use is usually not affected by the time passed from injury to surgery.

Neurolysis is performed through the popliteal approach when PN continuity is preserved. After skin incision, soft tissue dissection was performed to reveal and identify the PN. External neurolysis is performed when intraoperative findings showed that the nerve was compressed by surrounding scar/fibrous tissue. In situations when intraneural fibrosis was found, internal neurolysis is performed. After satisfactory deliberation of the nerve, hemostasis is performed and the wound is closed in a layered fashion. Usually, drainage was not needed.

The same popliteal approach is used for nerve repair as well, but, in situations when nerve continuity was not preserved. After identification and preparation of proximal and distal ends of PN, either direct (suture) or repair with various graft types was performed.

Direct repair (with 9/0 interrupted sutures) is possible when the nerve defect was less than 3 cm in length and adequate coaptation without tension could be achieved. When a longer nerve defect is found, it was repaired with sural nerve grafts, usually from the same-sided leg. Cable grafting was performed when the nerve defect was proximal to the ending branches and interfascicular autografting when the nerve defect included ending branches. The wound is then closed in a layered fashion.

For TTs, we usually used the tibialis posterior muscle tendon, which is divided into two slips. One slip is attached to the tibialis anterior muscle and the second one to the extensor digitorum longus muscle and extensor hallucis longus muscle. Suturing is performed with 2/0 sutures, followed by hemostasis and wounds closure in a layered fashion. Immobilization with above knee cast, and foot and fingers in extension, is set and kept for 6 weeks postoperatively.

After the surgery and wound healing (and plaster removal in patients with TTs), all patients were referred to physical treatment in dedicated rehabilitation centers and local health service providers for at least 6 months.

Follow-up included neurological examinations by an independent neurologist, and also functional assessment: monthly, during the first 3 months, and every 3 months later on. Postoperative recovery was recorded with MRC and the residual pain was graded on VAS of pain. The use of orthosis or walking aid was also recorded.

QoL evaluation was performed when no further recovery was expected. Since there is no dedicated tool to asses QoL in patients with PN injuries, we opted for the use of three questionaries including the Ulm questionnaire as a dedicated tool for peripheral nerve injuries (23), the Short Form 36 (SF-36) health survey as a general QoL inventory (24), and Stanmore questionnaire focusing on foot-drop correction (25).

### Statistical analysis

In the case of normally distributed variables, mean and standard deviation are shown in the tables, while the differences are tested using the *t*-test; in case of two groups, and ANOVA in the case of more than two groups. For variables not falling under the normal distribution, median, minimum, and maximum values are reported in tables, while the differences between groups are tested using the Man–Whitney–Wilcoxon test, for two groups, or the Kruskal–Wallis test in case more than two groups are present. Categorical variables are presented by the number of observations and the percentage, while *χ*^2^ or Fisher test is used to compare frequencies between groups. All data were analyzed using R 3.4.2. [R Core Team (2017). R: A language and environment for statistical computing. R Foundation for Statistical Computing, Vienna, Austria].

This case series has been reported in line with the PROCESS Guideline (26).

## Results

This study included 51 patients: 30 (59%) were treated with neurolysis, 12 (23%) with nerve repair, and 9 (18%) with TT. Patients were mostly male (70%) with a median age of 41 (19–69) years old, with an urban residence (80%), and medium education (86%). All patients were Caucasian. Table 1 shows the sociodemographic characteristics and preoperative status of the patients by surgery class.

**Table 1:** Sociodemographic characteristics and preoperative status of patients by surgery class.

	All participants	Neurolysis	Nerve repair	Tendon transfer	*p*-value
	*N* = 51	*N* = 30	*N* = 12	*N* = 9	
Gender					0.112
Male	36 (70.6%)	18 (60.0%)	11 (91.7%)	7 (77.8%)	
Female	15 (29.4%)	12 (40.0%)	1 (8.3%)	2 (22.2%)	
Age	41.0 (19.0–69.0)	43.5 (19.0–69.0)	42.0 (20.0–62.0)	38.0 (24.0–53.0)	0.542
Residence					0.546
Rural	10 (19.6%)	5 (16.7%)	2 (16.7%)	3 (33.3%)	
Urban	41 (80.4%)	25 (83.3%)	10 (83.3%)	6 (66.7%)	
Education					NA
High school	44 (86.3%)	25 (83.3%)	11 (91.7%)	8 (88.9%)	
College	6 (11.8%)	5 (16.7%)	0 (0.0%)	1 (11.1%)	
University	1 (2.0%)	0 (0.0%)	1 (8.3%)	0 (0.0%)	
Nature of injury					NA
Trauma	38 (74.5%)	20 (66.7%)	12 (100.0%)	6 (66.7%)	
Iatrogenic	13 (25.5%)	10 (33.3%)	0 (0.0%)	3 (33.3%)	
Class of injury					NA
Primary	41 (80.4%)	23 (76.7%)	12 (100.0%)	6 (66.7%)	
Secondary	10 (19.6%)	7 (23.3%)	0 (0.0%)	3 (33.3%)	
Nerve continuity					NA
Yes	37 (72.5%)	30 (100.0%)	0 (0.0%)	7 (77.8%)	
No	14 (27.5%)	0 (0.0%)	12 (100.0%)	2 (22.2%)	
No. of assoc. injuries					NA
0	15 (30.0%)	6 (20.0%)	6 (50.0%)	3 (37.5%)	
1	25 (50.0%)	19 (63.3%)	4 (33.3%)	2 (25.0%)	
2	7 (14.0%)	4 (13.3%)	2 (16.7%)	1 (12.5%)	
3	2 (4.0%)	1 (3.3%)	0 (0.0%)	1 (12.5%)	
5	1 (2.0%)	0 (0.0%)	0 (0.0%)	1 (12.5%)	
MDS (preop.)					NA
S0	49 (96.1%)	28 (93.3%)	12 (100.0%)	9 (100.0%)	
S1	2 (3.9%)	2 (6.7%)	0 (0.0%)	0 (0.0%)	

MDS, Mackinnon–Dellon scale; NA, not available.

Characteristics of the surgery and postsurgical treatment, and of the Ulm questionnaire are presented in Table 2. The majority of patients reported that they had experienced an improvement due to the surgery (63%), that they were satisfied with the results of the surgical treatment (76%), most would undergo the surgery again if they had known the results (88%), and just above one-third of patients noticed a significant pain relief (37%).

**Table 2:** Surgery characteristics and results of the Ulm questionnaire by surgery class.

	All participants	Neurolysis	Nerve repair	Tendon transfer	*p*-value
	*N* = 51	*N* = 30	*N* = 12	*N* = 9	
Timing of surgery (months)	4.5 (1.5–349.5)	4.5 (1.5–14.8)	3.0 (1.5–7.6)	18.6 (5.2–349.5)	<0.001
Physical treatment	6.0 (0.0–60.0)	6.0 (0.0–60.0)	6.0 (0.0–24.0)	4.0 (1.0–18.0)	0.414
Supplements					0.211
No	3 (5.9%)	1 (3.3%)	2 (16.7%)	0 (0.0%)	
Yes	48 (94.1%)	29 (96.7%)	10 (83.3%)	9 (100.0%)	
Orthosis					0.030
Preop.	8 (22.9%)	4 (23.5%)	3 (33.3%)	1 (11.1%)	
Postop.	13 (37.1%)	10 (58.8%)	2 (22.2%)	1 (11.1%)	
Preop./Postop.	14 (40.0%)	3 (17.6%)	4 (44.4%)	7 (77.8%)	
Did anything improve due to surgery?					0.435
Not at all	3 (5.9%)	1 (3.3%)	2 (16.7%)	0 (0.0%)	
Slightly	6 (11.8%)	2 (6.7%)	3 (25.0%)	1 (11.1%)	
Moderate	10 (19.6%)	5 (16.7%)	2 (16.7%)	3 (33.3%)	
Quite a bit	11 (21.6%)	7 (23.3%)	2 (16.7%)	2 (22.2%)	
Very much so	21 (41.2%)	15 (50.0%)	3 (25.0%)	3 (33.3%)	
How satisfied are you with the result of surgery?					0.157
Not at all	4 (7.8%)	1 (3.3%)	3 (25.0%)	0 (0.0%)	
Only slightly	4 (7.8%)	2 (6.7%)	1 (8.3%)	1 (11.1%)	
Moderately	4 (7.8%)	2 (6.7%)	2 (16.7%)	0 (0.0%)	
Quite a bit	11 (21.6%)	6 (20.0%)	1 (8.3%)	4 (44.4%)	
Very satisfied	28 (54.9%)	19 (63.3%)	5 (41.7%)	4 (44.4%)	
If you know the current result, would you undergo the procedure again?					0.476
Yes, without any doubt	36 (70.6%)	22 (73.3%)	7 (58.3%)	7 (77.8%)	
Yes, very likely	9 (17.6%)	4 (13.3%)	3 (25.0%)	2 (22.2%)	
No most likely not	3 (5.9%)	1 (3.3%)	2 (16.7%)	0 (0.0%)	
No certainly not	3 (5.9%)	3 (10.0%)	0 (0.0%)	0 (0.0%)	
Did your pain change since surgery?					0.063
Not at all	8 (15.7%)	1 (3.3%)	4 (33.3%)	3 (33.3%)	
Slightly	12 (23.5%)	6 (20.0%)	2 (16.7%)	4 (44.4%)	
Moderately	12 (23.5%)	9 (30.0%)	2 (16.7%)	1 (11.1%)	
Quite a bit	15 (29.4%)	10 (33.3%)	4 (33.3%)	1 (11.1%)	
Very much so	4 (7.8%)	4 (13.3%)	0 (0.0%)	0 (0.0%)	

**Figure 1:**
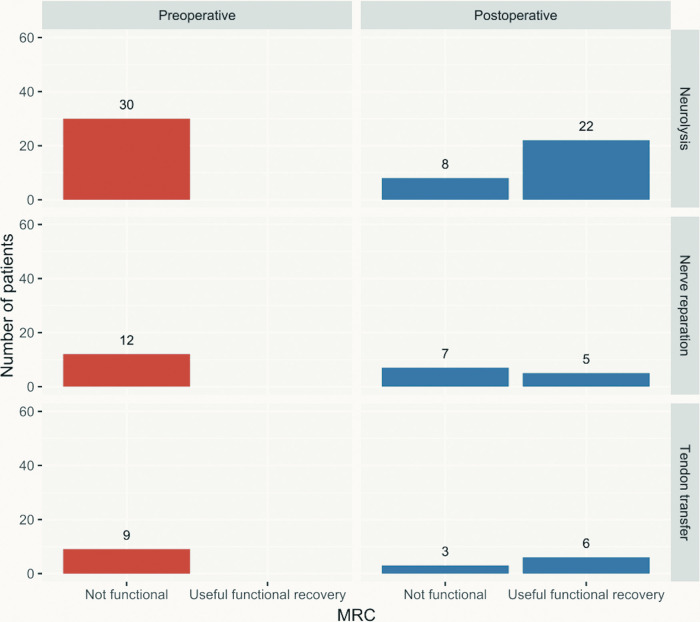
Preoperative vs. postoperative useful functional recovery according to the MRC scale by surgical technique applied. MRC, Medical Research Council.

Figure 1 shows the comparison between the preoperative and postoperative functional status according to the MRC scale. Thirty-three of the 51 included patients achieved useful functional recovery (M4 or M5), and 18 remained without significant improvement (*p* < 0.001) (≦M3). Neurolysis yields good results with an M3+ ratio nearing 80%, while useful functional recovery was achieved in 22 (73.3%) of 30 patients. Nerve repairs were followed by 58.33% of patients achieving M3+, while 41.66% recovered to a useful functional state. With the use of TT, all patients recovered to the M3+, while 66.7% recovered to M4.

**Figure 2:**
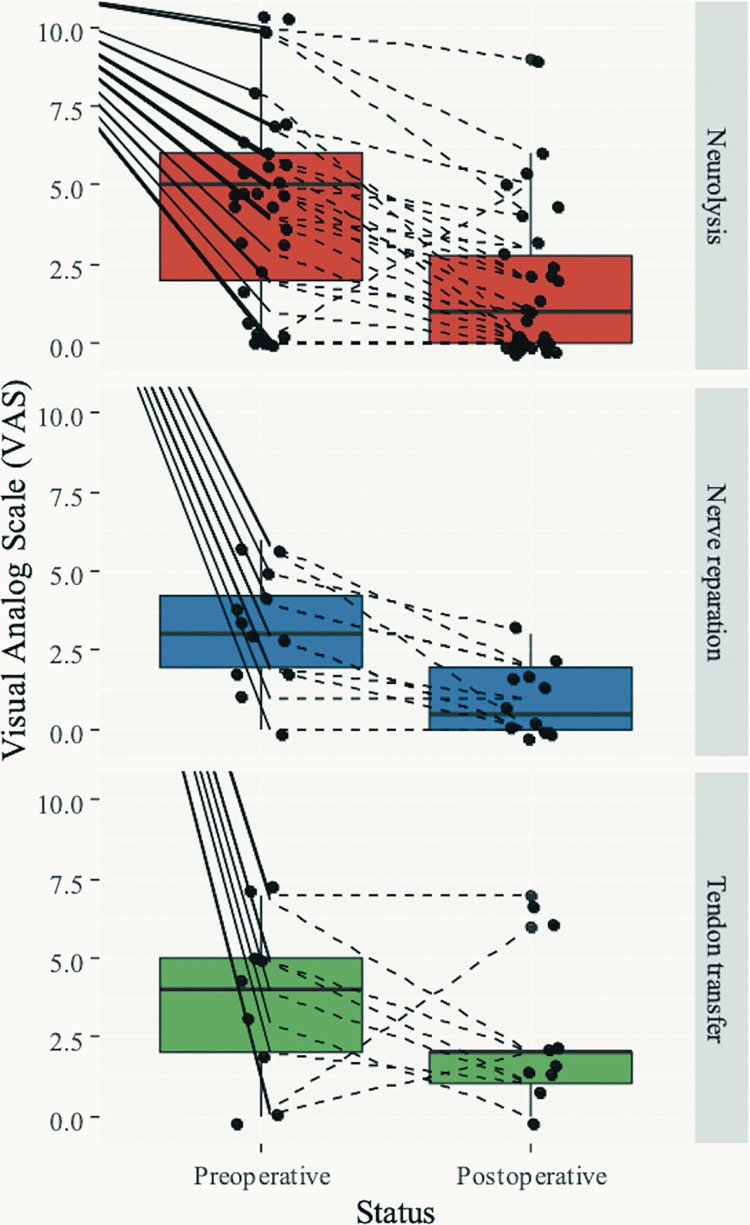
VAS pain scale difference between preoperative and postoperative status by class of surgery. VAS, visual-analog scale.

Figure 2 shows the differences between the preoperative and postoperative VAS in patients treated with different surgical approaches. The dashed lines represent the difference in single patients. Overall, in the majority of patients, the pain intensity reduced significantly; although, in each class of surgery, there were cases where the pain remained the same or even increased. The reduction in pain, as a difference in VAS scores, was statistically significant [Friedman test, *χ*^2^_(1) _= 31.8, *p* < 0.001].

**Figure 3:**
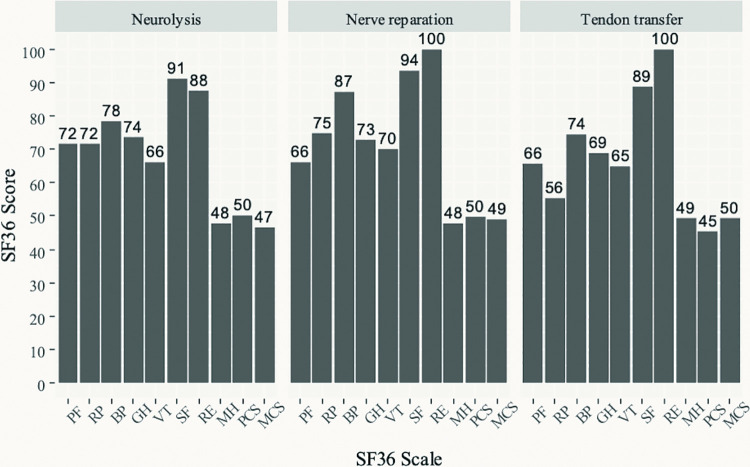
Results of the SF-36 quality of life questionnaire.

Figure 3 shows the results of the SF-36 QoL questionnaire in the three groups of patients. The highest (best) scores across the three groups are seen in the social functioning (SF) and role-emotional (RE) scales, followed by pain index (BP). Lower scores are seen on the physical functioning (PF), role-physical (RP), general health (GH) perceptions, and vitality (VT) scales. The lowest scores are seen on the mental health (MH) scale. Standardized physical component scales had values of 50 for neurolysis and nerve reparation, and 45 for TT, while for the standardized mental component scales the values were 47, 49, and 50, for neurolysis, nerve reparation and TT, respectively.

**Figure 4:**
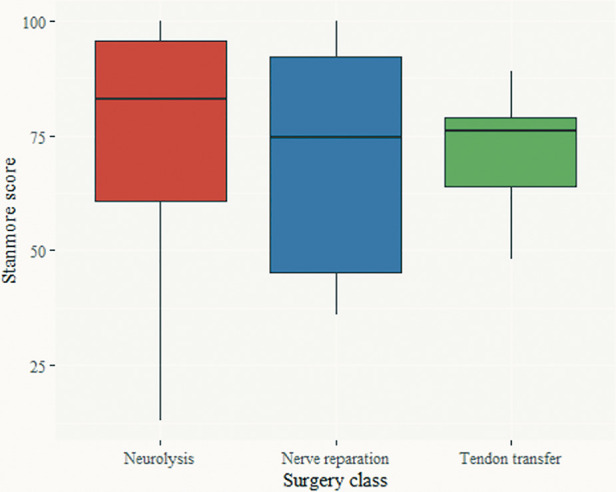
Stanmore scores among different surgical treatment modalities.

**Figure 5:**
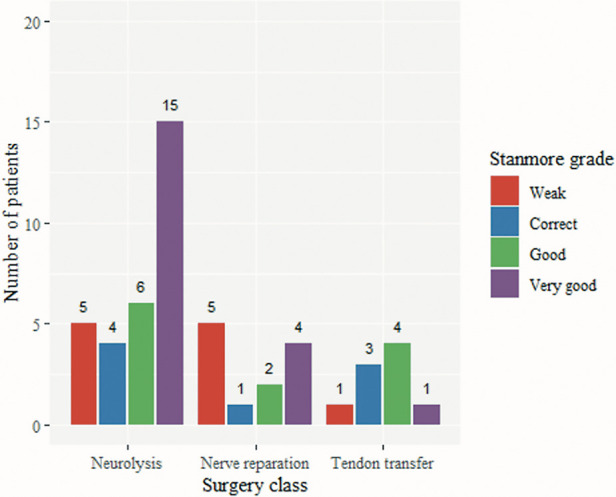
Stanmore grades among different surgical treatment modalities.

Figure 4 shows the Stanmore score by surgery class. There was no statistically significant difference in the Stanmore score (ANOVA, *F* = 0.419, *p* = 0.66), or the Stanmore grades (Figure 5) (weak, correct, good, and very good) among the three surgical treatment modalities.

## Discussion

The study evaluated the outcomes and QoL of 51 patients with PN injuries, who received one of three surgical treatment options (neurolysis, nerve repair, or TT), based on preoperative characteristics and individually tailored approaches.

PN injuries require surgical treatment in approximately 2/3 of cases (6), with the usual indication for surgery being more than 3 months after injury without recovery for closed injuries, and immediate or as soon-as-possible repair for open (sharp injuries or lacerations) (27). Timing for surgery is established as the most important factor that predisposes the recovery potential and also plays a major role in the choice of surgical technique (*p* < 0.001), together with the nerve continuity status and nature of injury (28).

These same principles were applied to our patients, although we have no data on patients who recovered or started to recover during the 3 months period, as these were not referred to the neurosurgical department. On the other hand, some patients reported for an initial exam when the reinnervation capacity was lost (more than 12 months without recovery after the injury, and with obvious target muscles atrophy). In these cases, we opted for TT rather than palliative bracing to achieve functional restoration (18).

The use of TT increased the percentage of surgically treated patients, but it is possible that it justified the rates for those patients who were not treated on time due to referral issues. The use of TT in patients with failed recovery after neurolysis or nerve repair is advised as a salvage procedure, although in our study, no patients underwent this kind of augmentation (29).

Neurolysis is employed in the least extensive nerve injuries when nerve continuity is preserved and yields good results with a motor recovery ratio nearing 80% (30, 31). These results correspond to ours, with 88% of patients achieving M3+, and 72.2% recovering to the M4+.

Nerve repairs of PN lesions were previously reported to have a roughly half (50%) motor recovery rate with the use of grafts, while the direct repair carries a somewhat higher rate of 60%–80% (30–32), in our study there was a slight increase as 58.33% of patients achieved M3+ recovery, while 41.66% recovered to the useful functional state with M4 and M5. It should be mentioned that 10 of 12 patients who underwent nerve repair received sural nerve grafts for the repair, one patient's nerve was directly sutured and one patient received an artificial conduit. Since the patients with nerve repair achieve satisfactory outcomes in roughly half of cases, it was proposed that these patients may undergo TT as a salvage procedure (12), and some authors even proposed to perform the one-stage nerve repair and TT immediately (33).

TT have very good results when only motor strength recovery is observed with recovery rates over 80% (almost 85% when concurrent posterior tibial TT was employed in a systematic review, and up to 100% in single studies (16, 31). However, this procedure is indicated as salvage, for isolated PN palsy with good ankle mobility, good strength of the posterior tibial muscle and poor prognosis of spontaneous recovery in order to decrease dependence on brace for walking, and to improve hip and knee function with improved gait kinematics (16, 34). All our patients recovered to the M3+, while 66.7% recovered to M4, but there were no cases who recovered to M5 which corresponds to the results of previous studies.

Pain was not an indication for surgery in our patients, but the common pattern of pain decrease was noted regardless of the surgical strategy. Previously, the authors have reported performing (internal or external) neurolysis to treat neuropathic pain (29, 35), especially in patients with gunshot wounds (36). Based on our results, we can hypothesize, that the origin of pain in our patients was not neuropathic in the majority, but rather chronic foot pain due to the instability and the arch flattening.

The most dedicated foot drop inventories focus on the use of bracing as a main aim of the treatment, and the use of TT, rather than the functional recovery following nerve release or repair, and actual QoL (20, 21, 37). Although in a relatively small cohort, there were no statistically significant differences in the QoL scores between the three treatment options, suggesting that no surgical technique influences the QoL by itself, but rather the right approach allows to achieve a similar QoL. A previous study discovered that patients with chronic foot drop had a reduced QoL with significantly poorer scores in the physical and psychosocial domains (38). This was not the case in our study, as the majority of patients recovered satisfactorily, leading to better overall scores.

While results from the three questionnaires focused on the overall QoL are consistent, when employing the Stanmore system, and assessing purely motor outcomes and the need for prosthesis, we found an apparent advantage of neurolysis, over nerve repair, over TT. Probably, due to the different regeneration potential, but also nerve injury severity, leading to favorable outcomes, compared to the previously reported 69% of patients with chronic foot drop in the need for bracing (38).

The insufficient number of patients (for a more powerful statistical analysis) overall, and especially in the TT and nerve repair groups is a usual limitation of studies on peripheral nerve injuries, and it is similar or even advantageous to other studies on the topic (16, 30–32).

There is no specific tool for the evaluation of QoL in patients with PN injuries, but we have shown that readily available tools can capture the QoL well, and quite consistently.

Future studies should focus on the improvement of all three surgical procedures, and a unified guided surgical decision-making process, as every procedure has its place in specific patients. Larger cohorts of patients should be recruited in a multidisciplinary fashion and merged within prospective trials leading to high-quality recommendations and guidelines.

## Conclusion

There is an apparent advantage of neurolysis, over nerve repair, over nerve transfer procedure, both in terms of useful functional recovery, and foot-drop-related QoL. However, when involving all aspects of QoL, these advantages diminish.

Individual approach to patients with severe PN injuries, involving all features and aspects of both injury and the patient, leads to the achievement of optimal results in all groups of patients, regardless of the regeneration potential and injury severity, but these should be considered as primary guides in choosing the surgical approach.

QoL system focusing on the peripheral nerve injuries is detrimental to a better understanding of the actual patient's state, recovery and satisfaction as present inventories lack specificity.

## Data Availability

The original contributions presented in the study are included in the article/Supplementary Material, further inquiries can be directed to the corresponding author/s.
